# Giant photon momentum locked THz emission in a centrosymmetric Dirac semimetal

**DOI:** 10.1126/sciadv.add7856

**Published:** 2023-01-04

**Authors:** Liang Cheng, Ying Xiong, Lixing Kang, Yu Gao, Qing Chang, Mengji Chen, Jingbo Qi, Hyunsoo Yang, Zheng Liu, Justin C.W. Song, Elbert E. M. Chia

**Affiliations:** ^1^ School of Electronic Science and Engineering, University of Electronic Science and Technology of China, Chengdu 610054, China.; ^2^ State Key Laboratory of Electronic Thin Films and Integrated Devices, University of Electronic Science and Technology of China, Chengdu 610054, China.; ^3^ Division of Physics and Applied Physics, School of Physical and Mathematical Sciences, Nanyang Technological University, Singapore 637371, Singapore.; ^4^ Division of Advanced Materials, Suzhou Institute of Nano-Tech and Nano-Bionics, Chinese Academy of Sciences, Suzhou 215123, China.; ^5^ Department of Electrical and Computer Engineering, National University of Singapore, Singapore 117576, Singapore.; ^6^ School of Materials Science and Engineering, Nanyang Technological University, 50 Nanyang Avenue, Singapore 637371, Singapore.

## Abstract

Strong second-order optical nonlinearities often require broken material centrosymmetry, thereby limiting the type and quality of materials used for nonlinear optical devices. Here, we report a giant and highly tunable terahertz (THz) emission from thin polycrystalline films of the centrosymmetric Dirac semimetal PtSe_2_. Our PtSe_2_ THz emission is turned on at oblique incidence and locked to the photon momentum of the incident pump beam. Notably, we find an emitted THz efficiency that is giant: It is two orders of magnitude larger than the standard THz-generating nonlinear crystal ZnTe and has values approaching that of the noncentrosymmetric topological material TaAs. Further, PtSe_2_ THz emission displays THz sign and amplitude that is controlled by the incident pump polarization and helicity state even as optical absorption is only weakly polarization dependent and helicity independent. Our work demonstrates how photon drag can activate pronounced optical nonlinearities that are available even in centrosymmetric Dirac materials.

## Teaser

We achieve strong THz emission with giant conversion efficiencies in acentrosymmetric Dirac semimetal.

## INTRODUCTION

The nontrivial winding of Bloch wave functions, encoded in its Bloch band quantum geometry and topology, plays a critical role in enhanced nonlinear responses (*1*). A case in point are topological semimetals, such as Weyl semimetals, which have recently become a fertile venue for realizing a variety of nonlinear optical effects (*2*, *3*). These include a giant second harmonic generation (*4*, *5*), large and near-quantized rectified photocurrents (*6*–*8*), and nonlinear Hall effects (*9*–*11*) even in the presence of time-reversal symmetry. These pronounced optical nonlinearities operate at ultrafast time scales and are poised for use in ultrafast technologies that include terahertz (THz) emission sources, as well as fundamental probes of electronic dynamics across the femtosecond-to-picosecond time scale (*12*–*16*).

Despite the large nonlinear rectified photocurrents (and associated THz emission) available in topological semimetals (*6*–*8*, *17*–*19*), there are limits to their practical implementation. For instance, second-order nonlinearities require broken material centrosymmetry, limiting the range of materials that can be used for nonlinear optical devices. Second, the rectified photocurrents/THz emission adopt a well-defined directionality fixed by its crystalline inversion asymmetry. As a result, they are expected to be scrambled in polycrystalline systems with randomized grains, and one needs to grow large, high-quality, single-crystalline systems for maximized THz emission efficiency. Last, nonlinear rectified photocurrents in noncentrosymmetric materials can be blind to the propagation direction and photon momentum (PM) of incident light. As a result, the photon momentum information of the incident light is often lost, hampering the development of photodetectors capable of sensing all the attributes of an incident beam of light (*20*).

**Fig. 1.**
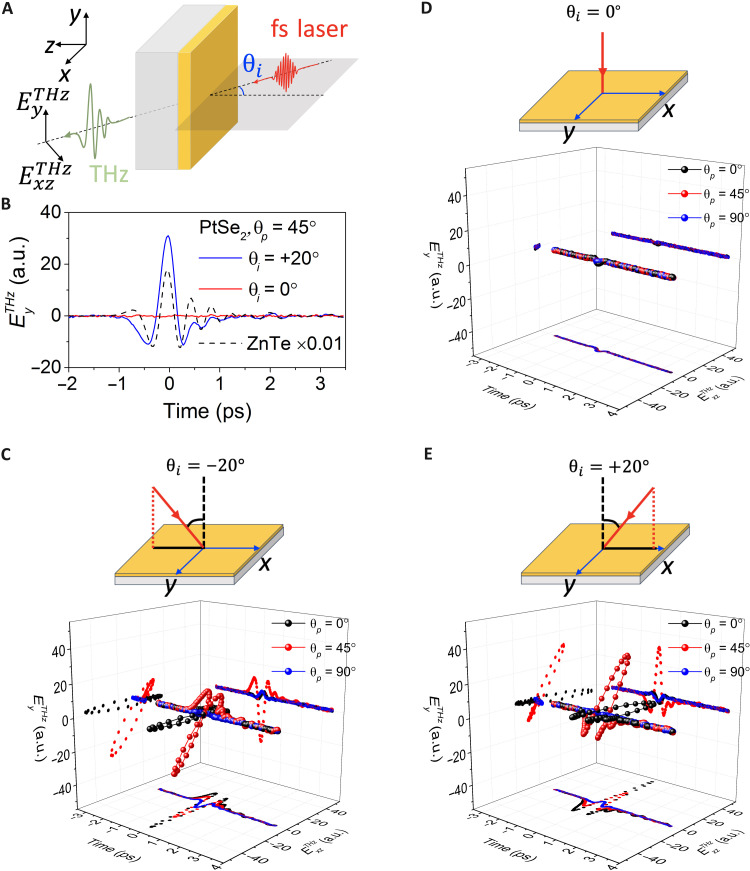
THz emission from PtSe_2_ in transmission mode. (**A**) Schematic of THz emission setup (for linear-polarized pump). Here, the *xyz* coordinate system represents the sample surface frame, and θ*_i_* is the incident angle, which is always on the *x*-*z* plane in the measurement (it is controlled by rotating the sample about the *y* axis, θ*_i_* = 0° manifests the normal incident pump). (**B**) Detected THz emission signal ($E_{y}^{THz}$ component) from the PtSe_2_ thin film as a function of time delay under the linear laser pump (θ*_p_* = 45°) with θ*_i_* = 0° (red curve) and +20° (blue curve), compared to the signal from the 0.5-mm-thick ZnTe crystal (dashed black curve). a.u., arbitrary units. (**C** to **E**) THz E-field ($E_{xz}^{THz}$ and $E_{y}^{THz}$) with selected incident pump angle θ*_i_* and polarization angle θ*_p_* (θ*_p_* = 0° manifests the horizontal polarization of the incident pump).

Here, we report a giant and highly tunable THz emission from thin polycrystalline films of the centrosymmetric Dirac semimetal PtSe_2_ upon fs-laser pulse excitation; the THz emission is turned on rapidly at oblique incidence. Unexpectedly, emitted THz amplitude per unit sample thickness (THz emission efficiency) is two orders of magnitude larger than that of the standard THz-generation nonlinear crystal ZnTe, with an efficiency that approaches those of the record-setting topological material TaAs (*4*, *12*, *13*). The emitted THz electric field (E-field) not only is directed along the incident plane (defined by incident pump light’s photon momentum PM) but also has large contributions transverse to PM (“*y*” direction) (see Fig. 1A). Notably, we find that THz emission is locked to the polarization state (PS) and incident direction: THz E-field sign, amplitude, and direction are fully controlled by incident pump polarization and helicity (i.e., its polarization state) as well as the incident angle.

As we discuss below, giant THz emission in our PtSe_2_ sample is a notable signature of the photon-drag effect (PDE) wherein PM transfer defines a symmetry-breaking axis (*21*–*29*). As we show, PM transfer works in concert with the incident PS to determine the directionality of giant THz emission. Furthermore, the THz emission in our sample exhibits very strong PS control even when absorption polarization dependence is weak. Perhaps the most notable are the large THz emission efficiencies of our THz emission: They far surpass previously reported photon-drag THz emission from other centrosymmetric systems, as will be discussed below. Our work demonstrates how photon drag can activate a rich and pronounced directional optical nonlinearity that are available even in centrosymmetric and polycrystalline Dirac materials.

## RESULTS

Experimentally, the multilayer polycrystalline PtSe_2_ thin film was fabricated on a sapphire substrate, with a thickness of ~50 nm and a roughness of ~10 nm measured by atomic force microscopy. Raman spectroscopy, x-ray diffraction measurement, transmission electron microscopy, and THz time-domain spectroscopy (THz-TDS) reveal good sample quality, consistent with previous reports (*30*, *31*); Drude model fitting shows the sample to be highly conducting, consistent with its multilayer nature (*31*, *32*) (see the sample characterization in the Supplementary Materials).

The schematic of THz emission measurements (in the transmission configuration) to investigate the transient photocurrent response of the PtSe_2_ thin film sample is shown in Fig. 1A, where the sample is excited by an 800-nm fs laser at oblique incidence. The incident angle (θ*_i_*) of the pump pulses is controlled by rotating the angle of the sample about the *y* axis as shown in Fig. 1A. Both the *y* and *xz* components of the emitted THz E-field are measured. When θ*_i_* ≠ 0°, $\$E_{xz}^{THz}\$$ contains component in both the *x* and *z* directions. In contrast to many other topological and two-dimensional materials, there is no observable THz emission signal for normal incidence (θ*_i_* = 0°, see Fig. 1B). This is consistent with the centrosymmetric crystal structure of PtSe_2_ (*33*, *34*), which typically prevents second-order nonlinear optical effects.

However, as shown in Fig. 1B, when an oblique incident pump (incident angle of θ*_i_* = + 20°) is used, we observe a strong THz emission from the sample under linear-polarized laser excitation ($\$E_{y}^{THz}\$$ component, linear pump polarization with θ*_p_* = 45°). Notably, we find that the emitted THz signal efficiency is larger than the typical THz generation crystals with broken centrosymmetry. As an illustration, Fig. 1B compares the THz signal of the PtSe_2_ thin film (50-nm thickness) with 0.5-mm-thick ZnTe with the same pump power and spot size. We observe that the PtSe_2_ under oblique incidence has a THz emission efficiency (per unit thickness) two orders of magnitude larger than that of ZnTe, as will also be discussed below.

**Fig. 2.**
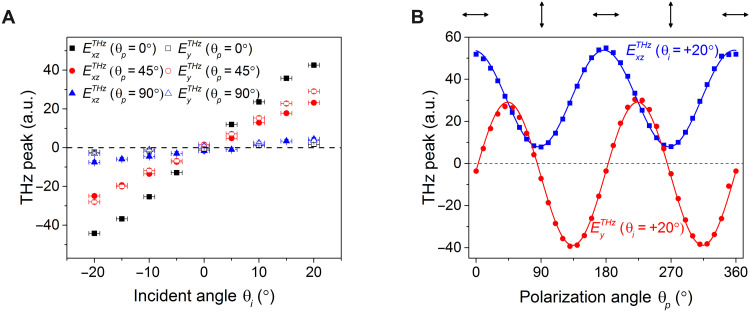
THz emission from the PtSe_2_ thin film under a pump laser with different linear polarization states. (**A**) Peak amplitude of emitted THz ($\$E_{xz}^{THz}\$$ and $\$E_{y}^{THz}\$$) from PtSe_2_ as a function of pump incident angle (θ*_i_*) with different polarization angles (θ*_p_*). (**B**) Pump polarization-dependent THz peak ($\$E_{xz}^{THz}\$$ and $\$E_{y}^{THz}\$$ components) for θ*_i_* = + 20°. The curves are from the sinusoidal fitting.

To clarify the origins of this giant and highly efficient THz emission from PtSe_2_, at oblique incidence, the THz emission signal with different incident angles and pump polarization angles is measured and analyzed (see in Fig. 1, C to E). We can clearly see that the polarity of THz waveform flips when the sign of incident angle changes (from θ*_i_* = − 20° to +20°), and there is negligible THz emission for normal incident pump (θ*_i_* = 0^°^). The key role of oblique incidence is exemplified in Fig. 2A where THz emission displays an almost linear dependence on incident angle across a different pump PS (across our measurement range). In addition, we note that the THz emission amplitude increases linearly with pump power (see fig. S3), indicating that the THz generation in our PtSe_2_ sample arises as a second-order nonlinear optical process.

Notably, the THz signal can be controlled by the PS of the pump laser: for instance, strong THz emission can be found for both linear pump polarization angles θ*_p_* = 0° and θ*_p_* = 45° but is suppressed for θ*_p_* = 90^°^. To tease out the strong polarization dependence of the THz emission of our PtSe_2_ sample, we continuously varied the polarization of the pump pulses θ*_p_* by tuning the half–wave plate (HWP) angle from 0° to 180^°^ (resulting in θ*_p_* ranging from 0^°^ to 360^°^), for an incident angle θ*_i_* at +20^°^. As shown in Fig. 2B, the emitted THz signal of the PtSe_2_ thin film is locked to the pump polarization angle θ*_p_*, evidenced by, for example, (i) the flip in sign of $\$E_{y}^{THz}\$$ (the *y* component of the emitted THz E-field, see Fig. 1A) when θ*_p_* changes from 45° to 135^°^, with almost zero $\$E_{y}^{THz}\$$ at θ*_p_* = 90^°^, (ii) maximal $\$E_{xz}^{THz}\$$ (the *xz* component of the emitted THz E-field, see Fig. 1A) at θ*_p_* = 0^°^ and almost zero $\$E_{xz}^{THz}\$$ at θ*_p_* = 90°. For both $\$E_{xz}^{THz}\$$ and $\$E_{y}^{THz}\$$, we find that the THz emission displays a sinusoidal dependence on θ*_p_* with a period of 180° but with $\$E_{y}^{THz}\$$ trailing $\$E_{xz}^{THz}\$$ by a phase difference of 45°. Similar data were obtained for θ*_i_* = − 20° but with the signs flipped compared to the θ*_i_* = + 20° case (see fig. S5A).

Comparing $\$E_{xz}^{THz}\$$ and $\$E_{y}^{THz}\$$ in Fig. 2B, we see that $\$E_{xz}^{THz}\$$ has a large nonzero offset, indicating a polarization-independent component of photocurrent in the *xz* direction. Nevertheless, the polarization-dependent part of $\$E_{xz}^{THz}\$$ is large and almost equal to the polarization-independent contribution, enabling the amplitude of $\$E_{xz}^{THz}\$$ to be tuned from maximum to almost zero by changing the pump polarization from θ*_p_* = 0^°^ to 90^°^ (Fig. 2B).

**Fig. 3.**
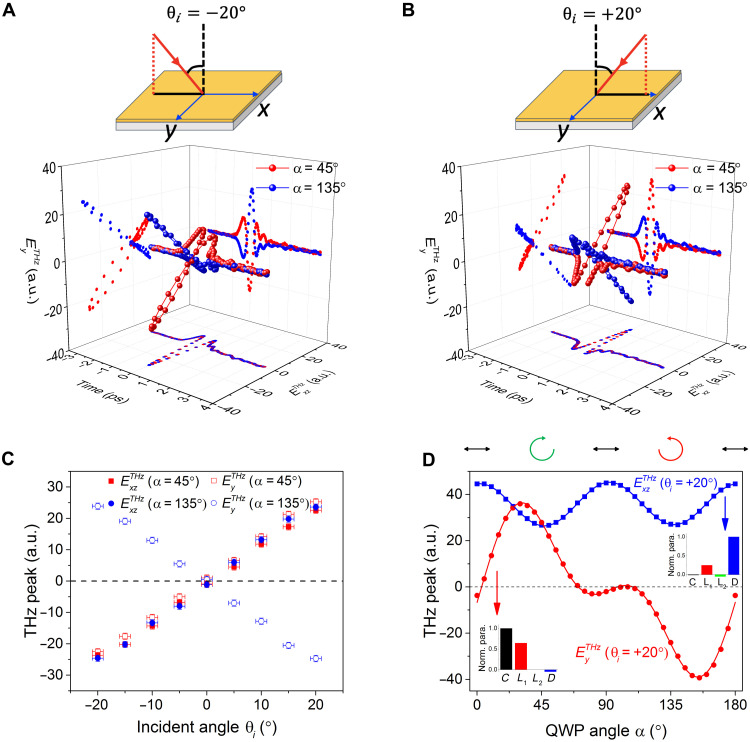
THz emission from the PtSe_2_ thin film under a pump laser with different helicities. (**A** and **B**) THz emission waveform from PtSe_2_ under a circular pump with different pump incident angles (θ*_i_* = ± 20°). (**C**) Peak amplitude of emitted THz ($\$E_{xz}^{THz}\$$ and $\$E_{y}^{THz}\$$) from PtSe_2_ as a function of incident angle with a left (α = 45°) and right (α = 135°) circular pump. (**D**) THz peak ($\$E_{xz}^{THz}\$$ and $\$E_{y}^{THz}\$$ components) as a function of QWP angle (α) for θ*_i_* = + 20°, and the curves are the fitting of Eq. 1. The insets in (D) are the normalized fitting parameters (α = 0° indicates that the fast axis of QWP is horizontal, which is the same as the polarization of incident laser before QWP).

To further understand how the THz emission depends on the PS, we investigated its helicity control using a quarter–wave plate (QWP) to tune the helicity of the pump laser from linear to circular polarization with the oblique incident pump. Figure 3 (A and B) shows the waveform of $\$E_{xz}^{THz}\$$ and $\$E_{y}^{THz}\$$ in the time domain, with a left (α = 45^°^) and a right (α = 135^°^) circular polarized pump. Similar to that discussed above, we observe a sign change of $\$E_{xz}^{THz}\$$ and $\$E_{y}^{THz}\$$ when the incident pump angle θ*_i_* is flipped (compare Fig. 3, A and B), and that under normal incidence, there is no THz emission. For a fixed θ*_i_*, the polarity of $\$E_{y}^{THz}\$$ flips when the pump is tuned from left to right circular polarization (with no change in the magnitude and time-domain waveform), whereas both the magnitude and polarity of $\$E_{xz}^{THz}\$$ are insensitive to the different circular pump polarization (see Fig. 3C comparing QWP angle α = 45° and 135^°^). Similar to that discussed above for linear polarization, both $\$E_{xz}^{THz}\$$ and $\$E_{y}^{THz}\$$ THz emission under circular polarization pump depend linearly on the incident pump angle θ*_i_* (Fig. 3C).

Locking to the helicity state of the pump is exemplified in Fig. 3D where both the sign and the magnitude of the THz peak can depend strongly on pump light helicity. While $\$E_{xz}^{THz}\$$ displays a sinusoidal dependence on the QWP angle α with a period of 180^°^, $\$E_{y}^{THz}\$$ exhibits a more complicated form. This latter dependence is consistent with the sign-changing helicity-dependent photocurrents along the *y* axis seen in Fig. 3 (A and B) and will be discussed below.

## DISCUSSION

Strong polarization (Fig. 2) and helicity (Fig. 3) dependence indicates the breaking of centrosymmetry and a preferred direction because photocurrent (associated with THz emission) is a vector. In contrast, our sample consists of a large-area (i) polycrystalline thin film consisting of randomly aligned grains of the (ii) centrosymmetric Dirac semimetal PtSe_2_. As a result, for our PtSe_2_ sample, the bulk is not expected to provide preferred directionality for THz emission.

**Fig. 4.**
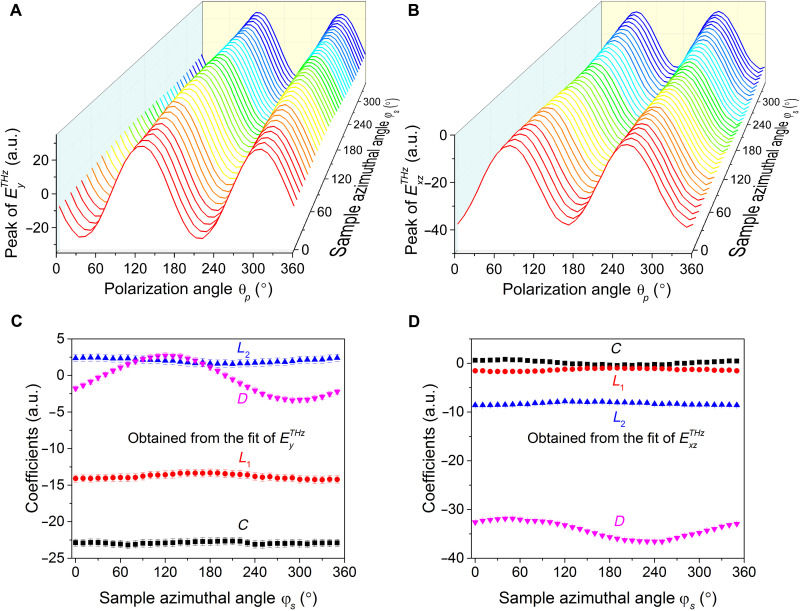
PM-locked THz emission at different sample azimuthal angles. (**A** and **B**) Peak amplitude of $\$E_{y}^{THz}\$$ and $\$E_{xz}^{THz}\$$ as a function of linear polarization angle θ*_p_* and sample azimuthal angle ϕ*_s_* (with an incident angle θ*_i_* = − 20°), respectively (**ϕ*_s_* = 0° manifests an arbitrary direction. Because the sample is polycrystalline, we cannot distinguish any special direction on the sample). (**C** and **D**) Fit parameters for $\$E_{y}^{THz}\$$ and $\$E_{xz}^{THz}\$$ components, respectively. The parameters are obtained from fitting QWP angle–dependent (α-dependent) THz peak data at different sample azimuthal angles ϕ*_s_* (fig. S4) to Eq. 1.

Instead, as we now argue, directionality is fixed by a combination of incident direction of the pump as well as its polarization state. To see this, we measure the THz emission signal as a function of azimuthal angle ϕ*_s_* (with the oblique incident angle θ*_i_* fixed). By varying ϕ*_s_*, the direction that the incident pump strikes the PtSe_2_ sample is effectively rotated about an axis perpendicular to the sample plane. Notably, as seen in Fig. 4 (A and B), along both $\$E_{y}^{THz}\$$ and $\$E_{xz}^{THz}\$$, linear polarization–dependent THz emission displays only very weak ϕ*_s_* dependence. This is consistent with the polycrystalline nature of our sample with no preferred orientation for the grains. The photocurrent generated is instead locked to the incident direction (i.e., PM) of the pump light.

A similar locking behavior (augmented by the PS of the pump) also manifests itself in the helicity-dependent THz emission. To see this, we first analyze THz emission dependence on QWP angle α quantitatively. This dependence can be fit by the phenomenological expression (see the Supplementary Materials)

$$E^{THz}=C\sin2\alpha +L_{1}\sin4\alpha +L_{2}\cos4\alpha +D$$ (1)

where the first term captures helicity-dependent THz emission, and the second and third terms describe THz emission that depend on linear pump polarization. The last term is a pump polarization–independent contribution. We fit our QWP THz emission data in Fig. 3D (θ*_i_* = + 20^°^) and fig. S5B (θ*_i_* = − 20^°^) to Eq. 1 (solid lines in the figures) with pronounced helicity and (linear) polarization-dependent contributions (see *C* and *L*_1_ in the inset of Fig. 3D). We note that as expected from Fig. 3 (C and D), helicity-dependent THz emission is most pronounced along $\$E_{y}^{THz}\$$ (characterized by the large and dominant *C* value in Fig. 4C), whereas $\$E_{xz}^{THz}\$$ THz emission is helicity-insensitive (characterized by the small *C* value in Fig. 4D). PM locking in these can be revealed in the same fashion as described above by rotating ϕ*_s_*. We find that the helicity-dependent photocurrents (see *C* coefficient for *E_y_* in Fig. 4C) are insensitive to ϕ*_s_*. This means that helicity-dependent photocurrents are locked to the *y* axis: They flow transverse to the PM.

The PM-locked photocurrents contrast with that of the conventional bulk photovoltaic effect (or from crystalline symmetry breaking at a surface) where photocurrent is expected to exhibit strong dependence on the crystal orientation and vanish in a polycrystalline sample (see also the Supplementary Materials). In the same vein, while a surface depletion field (oriented along the out-of-plane *z* direction) can induce classical drift currents along the out-of-plane direction (in the $\$E_{xz}^{THz}\$$ plane) (*35*, *36*) or even a third-order nonlinear Kerr-type effect (*37*), such fields are expected to be strongly screened in our metallic sample. We estimate the Thomas-Fermi screening length to be of order 2 nm (see the Supplementary Materials), far shorter than the penetration depth of 16 nm for the pump source used (see Table 1). As a result, we anticipate surface field effects to be severely quenched. Last, we note that other classical photocurrent effects such as the photo-Dember effect (*35*, *36*, *38*) or a photothermoelectric effect (*39*) are often polarization insensitive and flow in the out-of-plane direction, while our THz emission is highly PS sensitive and flow in both $\$E_{xz}^{THz}\$$ and $\$E_{y}^{THz}\$$ directions; we anticipate that such classical effects may contribute to the polarization-independent components of the observed signal along $\$E_{xz}^{THz}\$$.

**Table 1.** Comparison of THz emission efficiency between different typical materials. Here, $E_{\mathrm{peak}}^{THz}$ is the THz peak E-field, *F* is the pump fluence, *d* is the penetration depth for 800-nm light or the thickness of the sample, and η is the THz emission efficiency per length [$\eta =E_{\mathrm{peak}}^{THz}/(Fd)$].

Material	*E^THz^* (V/cm)	*F* (μJ/cm^2^)	*d* (nm)	θ*_i_* ( ° )	η (V/J)	Reference
Centrosymmetric						
PtSe_2_	23.5	283	16*	20	5.2 × 10^10^	This work
BSTS	3.2 × 10^−3^	340	27	22.5	3.5 × 10^6^	(*51*)
Multilayer graphene	7.0 × 10^−2^	35	14	25	1.4 × 10^9^	(*28*)
Vertical grown graphene	1.4	1410	1.5 × 10^3^	25	6.6 × 10^6^	(*25*)
Noncentrosymmetric						
TaAs	~600	2830	25	0	8.5 × 10^10^	(*12*)
ZnTe	982.6	283	5 × 10^5^	0	6.9 × 10^7^	This work
GaP	~50	127	2.5 × 10^5^	0	1.6 × 10^7^	(*52*)

*The penetration depth of PtSe_2_ is obtained from the fitting of reflectivity data (800-nm, s- and p-polarized light at different incident angle ranges from 5° to 90°) to the Fresnel equation, which is the same method as described in (*12*).

In contrast to the abovementioned mechanisms, the photocurrent and associated THz emission in our sample can be naturally explained by the PDE (*21*–*29*). Macroscopically, second-order nonlinear current requires breaking of centrosymmetry. In our system, this breaking can be naturally achieved by the combination of the PM and the PS of the pump light that control the axes for which photocurrents are aligned (*21*, *40*). When θ*_p_* = 0° or θ*_p_* = 90°, photon drag defines a preferred direction along *q* to produce $\$E_{xz}^{THz}\$$. Similarly, when a helical pump is used (or when θ*_p_* tilts away from vertical/horizontal polarizations), PM and PS can act together to produce strong $\$E_{y}^{THz}\$$ currents as well (see, e.g., the symmetry analysis of $\$E_{y}^{THz}\$$ from helicity-dependent pumps, section S4 in the Supplementary Materials), which is consistent with our observations (see Figs. 2 and 3).

We next turn to PS locking. What causes the strong PS-locked THz emission seen in the PtSe_2_ sample? In noncentrosymmetric (but time-reversal preserving) materials, strong THz emission and PS dependence are often attributed to quantum geometric photocurrents: Polarized irradiation results in shift and injection photocurrents (*1*, *7*, *41*–*44*). The former is associated with a real-space shift that electrons undergo upon a vertical interband transition (*1*, *7*, *41*, *42*, *44*), while the latter arises from a velocity difference the electron acquires as it is excited vertically from a lower to a higher band (*41*–*43*). In centrosymmetric materials, both these photocurrents vanish for vertical interband transitions. However, photon drag enables nonvertical shift and injection photocurrents (arising from a finite PM) to ensue (*21*, *40*): These photocurrents similarly arise from an electronic real-space shift and velocity changes an electron undergoes but now upon a nonvertical interband transition (that accounts for the incident PM). In both, PM plays a critical role in activating a finite photocurrent/THz emission response, allowing an asymmetric sampling of the interband transition matrix dipoles (intimately linked with quantum geometry) in momentum space (*41*–*43*).

This quantum geometric nature can induce strong PS-locked THz emission (*40*). To examine this, we analyze the PS-dependent reflectance of our PtSe_2_ sample at the same photon energy. Reflectance depends on the transition matrix elements integrated (averaged) across the interband transition contour in momentum space. Even as incident light polarization modulates the reflectance, reflectance oscillates with a maximum change of 20% as the linear polarization angle varies from θ*_p_* = 0° to 90° with the incident angle of 20° (fig. S7). Furthermore, we found no difference in reflectance between left and right circularly polarized light (see fig. S7B). In contrast, our THz emission displays strong PS locking: THz emission can be tuned from maximum to almost zero by changing the linear polarization (Fig. 2 from θ*_p_* = 0° to 90°) even as absorption at θ*_p_* = 90° is finite (as evidenced by our reflectance measurements). Further, the sign of photocurrent along $\$E_{y}^{THz}\$$ can be switched by helicity (as well as linear polarization) even as reflectance is the same for both left and right circularly polarized light. This contrast between reflectance and THz emission measurements provides strong evidence of the central role the momentum-dependent structure of the quantum geometry (e.g., interband transition matrix elements) plays in determining PS-locked THz emission.

Last, it is instructive to compare the efficiency of the THz emission from our PtSe_2_ sample with other materials including the benchmark nonlinear THz-generating (noncentrosymmetric) crystals such as ZnTe and TaAs. To do so, we compute the THz efficiency, defined as the THz peak per pump fluence per thickness (penetration depth) in Table 1. Notably, we find a giant THz efficiency for PtSe_2_ of 5.2 × 10^10^ V/J. PtSe_2_ THz efficiency outclasses that of the standard ZnTe by orders of magnitude and approaches that of the strong nonlinear crystal TaAs—both noncentrosymmetric materials. In comparison with other centrosymmetric materials, the THz efficiency of PtSe_2_ is larger than that previously reported in multilayered graphene by an order of magnitude. This demonstrates the giant nature of the PM- and PS-locked THz emission in our PtSe_2_ sample.

The highly efficient, PS- and PM-locked THz emission in PtSe_2_ sample demonstrates how PM can enable access to strong nonlinearities even in centrosymmetric materials rivaling that of record-setting noncentrosymmetric topological semimetals (*12*); PtSe_2_ is particularly attractive because its particle-hole asymmetric band structure (*33*, *45*) is expected to enhance photon drag (*40*, *46*). This high efficiency raises interesting prospects for nonlinear optoelectronics. For instance, using large THz emission activated by photon drag can provide a different modus operandi for THz emission: Instead of using high-quality and single-crystal noncentrosymmetric materials, polycrystalline centrosymmetric bulk materials (such as PtSe_2_) can be used to activate highly efficient THz signals controlled by the PM and PS of the incident pump. Similarly, when operated at steady state, we anticipate that the high efficiency we observe here can translate to large bulk DC photocurrents that may be used for highly sensitive and novel photodetectors, or when operated with a resistive load, bulk photovoltaics made from polycrystals.

## MATERIALS AND METHODS

### Sample preparation

PtSe_2_ films are synthesized via direct selenization of Pt-deposited sapphire substrates. First, Pt seeds of controlled thickness (35 nm) are deposited on sapphire with a thickness of 0.5 mm by an electron beam evaporator (Thermionics, VE-100). Then, Pt-deposited substrates are placed in the center zone of a horizontal quartz tube furnace (1 inch; Lindberg/Blue M Mini-Mite), and a quartz boat containing Se powder is placed at the furnace upstream side. After that, the system is purged with 300 standard cubic centimeters per minute (sccm) argon for 5 min to remove oxygen. Subsequently, the furnace is heated to the growth temperature of 550°C with a total ramping time of 30 min and is maintained for another 1 hour. During the growth reaction, the flow rate of Ar gas is maintained to be ∼100 sccm. After synthesis, the furnace is cooled to room temperature with the assistance of electric fans.

### THz emission measurement

THz emission spectroscopy of PtSe_2_ is measured by a homebuilt setup (*47*, *48*). The laser is a 1-kHz amplified laser with a wavelength of 800 nm and a pulse duration of 80 fs. The spot size on the sample is 1.5 mm. The emitted THz is collected by a pair of off-axis parabolic mirrors and focused on the THz detection crystal. THz detection is based on an electro-optical sampling technique, including a piece of 0.5-mm ZnTe crystal, QWP, Wollaston prism, and a balanced photodiode. Wiregrid THz polarizers are used to select and collect THz components in *y* and *xz* polarizations (see Fig. 1A). All the THz-related optics are enclosed in dry air environment with a humidity of less than 3%.

### THz time-domain spectroscopy

The THz-TDS of PtSe_2_ thin film is measured by TeraView TPS3000 in the frequency range of 0.3 to 3 THz. The transmitted THz from the sample (${\tilde{E}}_{\mathrm{samp}}^{THz}$, PtSe_2_ thin film on sapphire) and reference (${\tilde{E}}_{\mathrm{ref}}^{THz}$, sapphire), mounted on a sample holder with a clear aperture of 3.5 mm, is measured, and the complex THz conductivity $\tilde{\sigma }(\omega )$ that is extracted from the complex THz transmittance is $\tilde{T}(\omega )={\tilde{E}}_{\mathrm{samp}}^{THz}(\omega )/{\tilde{E}}_{\mathrm{refe}}^{THz}(\omega )$, and the data analysis method could be found in (*49*, *50*). The system is calibrated by measuring the transmittance of vacuum [${\tilde{T}}_{\mathrm{vac}}(\omega )$], which should be unity. In our system, the deviation of ${\tilde{T}}_{\mathrm{vac}}(\omega )$ from 1 is less than 0.5% in the frequency of 0.3 to 2.6 THz, indicating the trustable frequency window.

## Supplementary Materials

### This PDF file includes:

Supplementary Text Figs. S1 to S7 Table S1 References
