# Supplementary material for: Giant photon momentum locked THz emission in a centrosymmetric Dirac semimetal
Source: Sci Adv. 2023 Jan 4;9(1):eadd7856. doi: 10.1126/sciadv.add7856 (PMC9812375; doi:10.1126/sciadv.add7856)
Supplement: Supplementary file 1 — Supplementary Text Figs. S1 to S7 Table S1 References [file sciadv.add7856_sm.pdf]

Science Advances
AAAS

Supplementary Materials for

Giant photon momentum locked THz emission in a centrosymmetric Dirac semimetal

Liang Cheng *et al.*

Corresponding author: Justin C. W. Song, justinsong@ntu.edu.sg; Elbert E. M. Chia, elbertchia@ntu.edu.sg

Sci. Adv. **9**, eadd7856 (2023)
DOI: 10.1126/sciadv.add7856

This PDF file includes:

Supplementary Text
Figs. S1 to S7
Table 1
References

Supplementary Text

I. Sample characterization

AFM data

Figure S1A is a typical optical image of our $PtSe_2$ film, which exhibits uniform optical contrast. Atomic force microscopy (AFM) image (Figure S1B) reveals the resulted $PtSe_2$ film with a thickness about 50 nm.

Raman data

Fig. S1C shows the Raman spectrum of the $PtSe_2$ film revealing two feature peaks located at 173.4 cm^{-1} and 204.1 cm^{-1}, which correspond to E_g in-plane and A_{1g} out-of-plane Raman active modes, respectively. The positions of two Raman peaks are consistent with previous reports (*30, 31)*, and it reveals the good quality of the sample.

XRD data

The XRD pattern (Figure S1D) also confirms the high-quality of the resulting nanosheets with a hexagonal $P\bar{3}m1$ (164) space group. The main diffraction peaks correspond to the (001) planes of hexagonal $PtSe_2$ with a broad peak width and a small satellite peak, demonstrating the polycrystalline nature of the thin film.

TEM data

The TEM data (Figure S1E and F) shows that the grain size of our sample is 100-200 nm, and the orientation of the grains are randomly distributed. The inset in Fig. S1F demonstrates the selected area electron diffraction (SAED) patterns of $PtSe_2$, and the circles are assigned to (1 0 0), (0 1 1), (0 1 2), (1 1 0), (1 1 1) and (2 0 1) planes with lattice spacing of 3.22, 2.76, 1.96, 1.83, 1.72 and 1.52 Å, respectively.

THz time-domain spectroscopy data

Fig. S2 shows the THz conductivity extracted from THz transmission coefficient using standard THz data analysis *(50)*. The real part of the optical conductivity of the sample is very large and almost frequency-independent. The solid curves in Fig. S2 represent fits to the Drude model $[\tilde{\sigma}(\omega) = \epsilon_0\omega_p^2/(\gamma - i\omega) - i\epsilon_0\omega(\epsilon_\infty - 1)]$, where ω_p is the plasma frequency, γ is the scattering rate and ϵ_∞ is the high-frequency dielectric constant *(50)*. The fitting parameters are shown in Tab. S1, and their values are similar to typical metals *(49)*, suggesting the sample is in its highly conducting phase which is consistent with its multilayer nature *(31, 32)*. The good fits show that the Drude model is able to explain the conduction mechanism of $PtSe_2$, which is consistent with the fact that its Fermi level is far from the Dirac cone, that has been observed in ARPES measurements – the Dirac point is 1.2 eV below Fermi level for bulk $PtSe_2$. *(33, 34)*

II. Pump power dependence of THz emission

The dependence of THz emission on incident pump power is also measured with incident angle $\theta_i = +20°$, as see in Fig. S3. The good linearity of the dependence with different pump helicity (Fig. S3A) and linear polarization angle (Fig. S3B) indicates the second order nonlinear

nature of the observed THz emission. The weak deviation from linear fitting, especially at relatively higher power, suggests a little saturation absorption of $PtSe_2$ thin film.

III. Additional polarization/helicity dependent THz emission data

We also performed QWP-angle-dependent (α-dependent) THz peak measurements at different sample azimuthal angle (φ_s), which is used to obtain the CPDE parameters as function of α (Fig. 4C and D in main text). As seen in Fig. S4, the dependence of emitted THz peak on sample azimuthal angle φ_s is relatively weak, and the α-dependent trends in E_x^{THz} and E_y^{THz} do not change at different φ_s. In Fig. S5, the emitted THz peak as functions of polarization angle θ_p and QWP angle α with pump incident angle $\theta_i = -20°$ for E_{xz}^{THz} and E_y^{THz} are shown, which is with the same symmetry but opposite sign compared to the data for $\theta_i = 20°$ (Fig. 2B and 3D).The detailed discussion is in the main text.

IV. Symmetry analysis and fitting equation for α-dependence of photocurrents

In this section, we show that the bulk photovoltaic effect vanishes for $\boldsymbol{q} = 0$ when the material preserves inversion symmetry while the photon drag effect enabled by finite $\boldsymbol{q}$ is in general allowed. We then derive a general formula for the photon drag current as a function of the quarter waveplate angle α [Eq. (1) of the main text], which well describes the experimental data (Figure. 3D of the main text).

To begin with, we first consider a material irradiated by light with electric field $\boldsymbol{E^i}$. For $\boldsymbol{q} = 0$, the conventional bulk photovoltaic currents can be expressed as $j_a(\boldsymbol{q} = 0) = \sum_{b,c} \sigma_{abc} E_b^i \left(E_c^i\right)^*$. Under spatial inversion, both electric field $\boldsymbol{E}^i \to -\boldsymbol{E}^i$ and the photocurrent $\boldsymbol{j} \to -\boldsymbol{j}$ will reverse their directions. However, the nonlinear conductivity tensor is invariant under spatial inversion when the material is centrosymmetric. As a result, the bulk photovoltaic current vanishes in centrosymmetric materials.

However, when we consider the photon drag effect with $\boldsymbol{q} \neq 0$, the photocurrents (up to the first order of $\boldsymbol{q}$) can be written as $j_a = \sum_{b,c,d} \chi_{abcd} E_b^i \left(E_c^i\right)^* q_d$. Under spatial inversion, we have $\boldsymbol{q} \to -\boldsymbol{q}$, $\boldsymbol{E}^i \to -\boldsymbol{E}^i$ and $\boldsymbol{j} \to -\boldsymbol{j}$. Thus, σ_{abcd} and the photon drag currents can be nonzero in general even in centrosymmetric materials, where χ_{abcd} remains invariant under spatial inversion.

Next, we consider a material irradiated by light passed through a quarter waveplate and analyze the photocurrent dependence on the quarter waveplate angle α. As illustrated by Fig. S6, we consider light passing through a quarter wave plate irradiated onto the surface of sample with an incident angle θ_i. Here, we denote the lab frame as $x'y'z'$ and the sample frame as xyz, with $\hat{\boldsymbol{x}}' = \cos\theta_i\,\hat{\boldsymbol{x}} - \sin\theta_i\,\hat{\boldsymbol{z}}$, $\hat{\boldsymbol{y}}' = \hat{\boldsymbol{y}}$ and $\hat{\boldsymbol{z}}' = \cos\theta_i\,\hat{\boldsymbol{z}} + \sin\theta_i\,\hat{\boldsymbol{x}}$. The light wavevector is thus $\boldsymbol{q} = q\hat{\boldsymbol{z}}' = q(\cos\theta_i\,\hat{\boldsymbol{z}} + \sin\theta_i\,\hat{\boldsymbol{x}})$. For simplicity, we have fixed the sample azimuthal angle and write the nonlinear $\boldsymbol{q}$-dependent conductivity tensor χ_{abcd} in the sample frame.

In the experiment, a p-polarized light (with electric field $\boldsymbol{E}^i = E_0\hat{\boldsymbol{x}}' e^{-i\omega t + i\boldsymbol{q}\cdot\boldsymbol{r}}$) is passed through the quarter wave plate, which is perpendicular to the light propagating direction with its

fast axis rotated by angle α away from $\hat{\boldsymbol{x}}'$. The resulting electric field after passing through the quarter wave plate is thus given by

Eq. S1

$$\begin{aligned}\boldsymbol{E}^i &= E_0(\cos^2\alpha + i\sin^2\alpha)\hat{\boldsymbol{x}}' + E_0\sin\alpha\cos\alpha\,(1-i)\hat{\boldsymbol{y}}' \\ &= E_0\cos\theta_i\,(\cos^2\alpha + i\sin^2\alpha)\hat{\boldsymbol{x}} + E_0\sin\alpha\cos\alpha\,(1-i)\hat{\boldsymbol{y}} \\ &\quad - E_0\sin\theta_i\,(\cos^2\alpha + i\sin^2\alpha)\hat{\boldsymbol{z}} = E_x^i\hat{\boldsymbol{x}} + E_y^i\hat{\boldsymbol{y}} + E_z^i\hat{\boldsymbol{z}}\end{aligned}$$

To further understand the polarization and helicity dependence, we write $j_a = \sum_{b,c,d}\chi^{\mathrm{Re}}_{abcd}\mathrm{Re}[E_b^i(E_c^i)^*]q_d - \chi^{\mathrm{Im}}_{abcd}\mathrm{Im}[E_b^i(E_c^i)^*]q_d$, where j_a are the real photocurrents along $\hat{\boldsymbol{a}}$ in the lab frame, $\chi^{\mathrm{Re}}_{abcd}$ accounts for the photocurrents induced by linearly polarized light, while $\chi^{\mathrm{Im}}_{abcd}$ accounts for the helicity dependent circular photocurrents. For clarity, here we have expanded the photocurrents up to the first order in $\boldsymbol{q}$. By explicitly writing down all possible terms induced by the electric field in Eq. (S1), we obtain the photon drag induced photocurrents as a function of α:

Eq. S2

$$j_a = D^a + L_1^a\sin 4\alpha + L_2^a\cos 4\alpha + C^a\sin 2\alpha$$

where the coefficients D^a, L_1^a, L_2^a and C^a are

Eq. S3

$$\begin{aligned}D^a = \frac{1}{4}E_0^2 q\big[3(\chi^{\mathrm{Re}}_{axxx}\sin\theta_i + \chi^{\mathrm{Re}}_{axxz}\cos\theta_i)\cos^2\theta_i + \chi^{\mathrm{Re}}_{ayyx}\sin\theta_i + \chi^{\mathrm{Re}}_{ayyz}\cos\theta_i \\ + 3(\chi^{\mathrm{Re}}_{azzx}\sin\theta_i + \chi^{\mathrm{Re}}_{azzz}\cos\theta_i)\sin^2\theta_i - 3\big(\chi^{\mathrm{S,Re}}_{axzx}\sin\theta_i + \chi^{\mathrm{S,Re}}_{axzz}\cos\theta\big)\sin 2\theta_i\big]\end{aligned}$$

Eq. S4

$$L_1^a = \frac{1}{2}E_0^2 q\big[\big(\chi^{\mathrm{S,Re}}_{ayxx}\sin\theta_i + \chi^{\mathrm{S,Re}}_{ayxz}\cos\theta_i\big)\cos\theta_i - \big(\chi^{\mathrm{S,Re}}_{ayzx}\sin\theta_i + \chi^{\mathrm{S,Re}}_{ayzz}\cos\theta_i\big)\sin\theta_i\big]$$

Eq. S5

$$\begin{aligned}L_2^a = \frac{1}{4}E_0^2 q\big[(\chi^{\mathrm{Re}}_{axxx}\sin\theta_i + \chi^{\mathrm{Re}}_{axxz}\cos\theta_i)\cos^2\theta_i - \chi^{\mathrm{Re}}_{ayyx}\sin\theta_i - \chi^{\mathrm{Re}}_{ayyz}\cos\theta_i \\ + (\chi^{\mathrm{Re}}_{azzx}\sin\theta_i + \chi^{\mathrm{Re}}_{azzz}\cos\theta_i)\sin^2\theta_i - \big(\chi^{\mathrm{S,Re}}_{axzx}\sin\theta_i + \chi^{\mathrm{S,Re}}_{axzz}\cos\theta_i\big)\sin 2\theta_i\big]\end{aligned}$$

Eq. S6

$$C^a = E_0^2 q\big[\big(\chi^{\mathrm{A,Im}}_{ayxx}\sin\theta_i + \chi^{\mathrm{A,Im}}_{ayxz}\cos\theta_i\big)\cos\theta_i - \big(\chi^{\mathrm{A,Im}}_{ayzx}\sin\theta_i + \chi^{\mathrm{A,Im}}_{ayzz}\cos\theta_i\big)\sin\theta_i\big]$$

In the above, we have defined $\chi^{\mathrm{S}}_{a_0b_0c_0d_0} = \frac{1}{2}\big(\chi_{a_0b_0c_0d_0} + \chi_{a_0c_0b_0d_0}\big)$ and $\chi^{\mathrm{A}}_{a_0b_0c_0d_0} = \frac{1}{2}\big(\chi_{a_0b_0c_0d_0} - \chi_{a_0c_0b_0d_0}\big)$ as the symmetric and antisymmetric part of the nonlinear susceptibility tensor.

In the experiment, we have measured the THz emission E_y^{THz} associated with the photocurrent j_a and E_{xz}^{THz} associated with the photocurrent $\boldsymbol{j}\cdot\hat{\boldsymbol{x}}'$. Recalling that $\hat{\boldsymbol{x}}' = \cos\theta_i\,\hat{\boldsymbol{x}} - \sin\theta_i\,\hat{\boldsymbol{z}}$, the photocurrent leading to THz signals is given by $\boldsymbol{j}\cdot\hat{\boldsymbol{x}}' = j_x\cos\theta_i - j_z\sin\theta_i$, where j_x and j_y are photocurrents in the sample frame. Thus, the observed photocurrent follows

Eq. S7

$$\boldsymbol{j}\cdot\hat{\boldsymbol{x}}' = D + L_1\sin 4\alpha + L_2\cos 4\alpha + C\sin 2\alpha$$

where $D = D^x \cos\theta_i - D^z \sin\theta_i$, $L_1 = L_1^x \cos\theta_i - L_1^z \sin\theta_i$, $L_2 = L_2^x \cos\theta_i - L_2^z \sin\theta_i$, and $C = C^x \cos\theta_i - C^z \sin\theta_i$.

We note that here we have written done all possible contributions χ_{abcd} allowed by inversion symmetry without considering any additional point group symmetries. Since the sample we used is polycrystalline with multiple grains randomly oriented, it is not practical to identify any high symmetry axis or confirm any preferred crystal orientation. However, we note that not all terms in Eq. (S2-S7) are necessarily nonzero. Indeed, we have found that the THz signals reverse their sign with the incident angle $\theta_i \to -\theta_i$, thus the terms that are odd in θ_i in Eq. (S2-S7) dominate the photoresponse.

V. Symmetry analysis of surface photogalvanic effect in polycrystal

We consider a grain with crystal axis rotated away from the sample surface xyz frame by an azimuthal angle φ about the z-axis and angle ψ about the y-axis (z and y -axes are shown in Fig. S6). We denote the grain crystal frame as $x_0y_0z_0$. From xyz frame to $x_0y_0z_0$, the rotation matrix is given by

Eq. S8

$$R = \begin{pmatrix} \cos\psi\cos\varphi & \sin\varphi & \sin\psi\cos\varphi \\ \cos\psi\sin\varphi & \cos\varphi & \sin\psi\sin\varphi \\ -\sin\psi & 0 & \cos\psi \end{pmatrix}.$$

On the surface, the point group of $PtSe_2$ is reduced from D_{3d} (centrosymmetric) to C_{3v} (noncentrosymmetric) *(53)*, and the second-order nonlinear conductivity can be nonzero, leading to finite photocurrent $j_{a_0} = \sum_{b_0c_0} \sigma_{a_0b_0c_0} E_b^i \left(E_c^i\right)^*$. Note that here we have expressed the photocurrent, nonlinear conductivity tensor and electric field in the crystal frame $x_0y_0z_0$.

For C_{3v} group, the nonzero elements of the $\sigma^{(2)}_{a_0b_0c_0}$ tensor are *(54)*:

Eq. S9

$$\begin{cases} \sigma_{x_0z_0x_0} = \sigma_{y_0z_0y_0} \\ \sigma_{x_0x_0z_0} = \sigma_{y_0y_0z_0} \\ \sigma_{z_0x_0x_0} = \sigma_{z_0y_0y_0} \\ \sigma_{z_0z_0z_0} \\ \sigma_{y_0y_0y_0} = -\sigma_{y_0x_0x_0} = -\sigma_{x_0x_0y_0} = -\sigma_{x_0y_0x_0} \end{cases}.$$

We examine the photocurrent induced by surface symmetry breaking for an incident light passing through a quarter wave plate angle with its fast axis rotated by angle α away from the x' axis. The light electric field amplitude can be expressed as Eq. S1. In the $x_0y_0z_0$ frame, the electric field can be written as:

Eq. S10

$$\begin{pmatrix} E_{x_0}^i \\ E_{y_0}^i \\ E_{z_0}^i \end{pmatrix} = R \begin{pmatrix} E_x^i \\ E_y^i \\ E_z^i \end{pmatrix}.$$

Substituting the electric field (Eq. S1) and the nonlinear conductivity tensors (Eq. S9), we can obtain the photocurrent in the $x_0y_0z_0$ frame as:

Eq. S11

$$
\begin{aligned}
j_{x_0} = -E_0^2 \sigma^{\mathrm{S,Re}}_{x_0x_0z_0} \Big[& (\sin 2\psi \cos\varphi \cos 2\theta_i + \cos 2\psi \cos\varphi \sin 2\theta_i)(\cos^4\alpha + \sin^4\alpha) \\
& - \frac{1}{2}(\sin\psi \sin\varphi \cos\theta_i + \cos\psi \sin\varphi \sin\theta_i)\sin 4\alpha \Big] \\
& + E_0^2 \sigma^{\mathrm{S,Re}}_{x_0x_0y_0} \Big[\Big(\cos^2\psi \sin 2\varphi \cos^2\theta_i + \sin^2\psi \sin 2\varphi \sin^2\theta_i \\
& - \frac{1}{2}\sin 2\psi \sin 2\varphi \sin 2\theta_i \Big)(\cos^4\alpha + \sin^4\alpha) \\
& + \frac{1}{2}(\cos\psi \cos 2\varphi \cos\theta_i - \sin\psi \cos 2\varphi \sin\theta_i)\sin 4\alpha - \frac{1}{2}\sin 2\varphi \sin^2 2\alpha \Big] \\
& + E_0^2 \sigma^{\mathrm{A,Im}}_{x_0x_0z_0}(\sin\psi \sin\varphi \cos\theta_i + \cos\psi \sin\varphi \sin\theta_i)\sin 2\alpha \\
& - E_0^2 \sigma^{\mathrm{A,Im}}_{x_0x_0y_0}(\cos\psi \cos\theta_i - \sin\psi \sin\theta_i)\sin 2\alpha,
\end{aligned}
$$

and

Eq. S12

$$
\begin{aligned}
j_{y_0} = E_0^2 \sigma_{y_0x_0x_0} \Big[& \Big(\cos^2\psi \cos 2\varphi \cos^2\theta_i + \sin^2\psi \cos 2\varphi \sin^2\theta_i \\
& - \frac{1}{2}\sin 2\psi \cos 2\varphi \sin 2\theta_i \Big)(\cos^4\alpha + \sin^4\alpha) - \frac{1}{2}\cos 2\varphi \sin^2 2\alpha \\
& - \frac{1}{2}(\cos\psi \sin 2\varphi \cos\theta_i - \sin\psi \sin 2\varphi \sin\theta_i)\sin 4\alpha \Big] \\
& - E_0^2 \sigma^{\mathrm{S,Re}}_{y_0y_0z_0} \Big[(\sin 2\psi \sin\varphi \cos 2\theta_i + \cos 2\psi \sin\varphi \sin 2\theta_i)(\cos^4\alpha + \sin^4\alpha) \\
& + \frac{1}{2}(\sin\psi \cos\varphi \cos\theta_i + \cos\psi \cos\varphi \sin\theta_i)\sin 4\alpha \Big] \\
& - E_0^2 \sigma^{\mathrm{A,Im}}_{y_0y_0z_0}(\sin\psi \cos\varphi \cos\theta_i + \cos\psi \cos\varphi \sin\theta_i)\sin 2\alpha.
\end{aligned}
$$

Here, the superscript Re (Im) denote the real (imaginary) part of the nonlinear conductivity. Furthermore, in the same fashion as the previous section, we have also defined $\sigma^{\mathrm{S}}_{a_0b_0c_0} = \frac{1}{2}\left(\sigma_{a_0b_0c_0} + \sigma_{a_0c_0b_0}\right)$ and $\sigma^{\mathrm{A}}_{a_0b_0c_0} = \frac{1}{2}\left(\sigma_{a_0b_0c_0} - \sigma_{a_0c_0b_0}\right)$ as the symmetric and antisymmetric part of the nonlinear conductivity tensor.

We then project the photocurrents into the xyz frame by noting that the inverse rotation matrix

Eq. S13
$$
R^{-1} = \begin{pmatrix} \cos\psi\cos\varphi & \cos\psi\sin\varphi & -\sin\psi \\ -\sin\varphi & \cos\varphi & 0 \\ \sin\psi\cos\varphi & \sin\psi\sin\varphi & \cos\psi \end{pmatrix},
$$

and the photocurrent in the xyz frame is given by

Eq. S14
$$
\begin{pmatrix} j_x \\ j_y \\ j_z \end{pmatrix} = R^{-1} \begin{pmatrix} j_{x_0} \\ j_{y_0} \\ j_{z_0} \end{pmatrix}.
$$

The in-plane photocurrent transverse to the incidence plane is given by

Eq. S15

$$j_y = -\sin\varphi\, j_{x_0} + \cos\varphi\, j_{y_0}$$
$$= E_0^2\sigma_{y_0x_0x_0}\left[\cos 3\varphi\left(\cos^2\psi\cos^2\theta_i + \sin^2\psi\sin^2\theta_i - \frac{1}{2}\sin 2\psi\sin 2\theta_i\right)(\cos^4\alpha + \sin^4\alpha) - \cos 3\varphi\sin^2 2\alpha - \sin 3\varphi\,(\cos\psi\cos\theta_i - \sin\psi\sin\theta_i)\sin 4\alpha\right]$$
$$- \frac{1}{2}E_0^2\sigma_{y_0y_0z_0}^{S,Re}(\sin\psi\cos\theta_i + \cos\psi\sin\theta_i)\sin 4\alpha$$
$$- E_0^2\sigma_{y_0y_0z_0}^{A,Im}(\sin\psi\cos\theta_i + \cos\psi\sin\theta_i)\sin 2\alpha.$$

The photocurrent induced by surface symmetry breaking (as shown in Eq. S15) exhibit large azimuthal angle dependence. This contrasts with our experimental THz emission signal, which is largely azimuthal angle independent. Additionally, we note that our sample is polycrystalline with many randomly oriented grains. The effect of this polycrystallinity can be simulated by integrating the rotation angles ψ and φ from 0 to 2π. As a result, we find that the net j_y vanishes in a polycrystalline sample. This is in contrast with our experimental observation, whereby large THz emission signal E_y (resulting from photocurrent j_y) has been detected.

VI. Estimate of Screening Length in $PtSe_2$

In this section, we estimate the screening length in a polycrystal $PtSe_2$ sample by considering an isotropic effective mass with a range of values and calculating the corresponding range of Thomas-Fermi screening length.

From Ref. *(45)*, we consider an effective mass m_{eff} that ranges from $0.22m_e$ to $2.5m_e$, where m_e is the electron rest mass. We note that the material can be well described by Drude model, and the charge density can be estimated from the plasma resonance frequency $\omega_p = 2\pi \times 273\,\text{THz}$ obtained from the THz-TDS measurement (See the Note 1 and Table 1 in this Supplementary Materials). The carrier density can be obtained by

Eq. S16

$$n = \frac{\omega_p^2\varepsilon_0 m_{\text{eff}}}{q^2} = (0.2049 \sim 2.328) \times 10^{27}\ \text{m}^{-3}.$$

The density of the state is given by $g(\epsilon_F) = \sqrt{2m_{\text{eff}}^3\epsilon/\pi^2\hbar^3}$. The inverse of the Thomas-Fermi screening length k_s can be estimated by *(55)*:

Eq. S17

$$k_s = \sqrt{\frac{q^2 g(\epsilon_F)}{\varepsilon_0}},$$

where $\epsilon_F = \frac{\hbar^2}{2m_{\text{eff}}}(3\pi^2 n)^{2/3}$ is the Fermi energy. Substituting the effective mass and the corresponding carrier density, we obtain a range of the inverse Thomas-Fermi screening length$k_s = 3.1{\sim}16\,nm^{-1}$, whereas the Fermi wavevector is in the range $k_F = 2.2{\sim}7.5\,nm^{-1}$.

Since Thomas-Fermi screening is valid in the long wavelength limit, the strong screening we see above is cut by the electron wavelength $2\pi/k_F$. This yields a screening length $l_s = 0.84 \sim 2.8\ nm$ in our $PtSe_2$ sample.

We note that the screening length that is less than 3 nm, which is much smaller than both the penetration depth of the incident light (16 nm) and the roughness of the sample (10 nm). Thus, we expect the effects due to surface depletion field are strongly quenched in our sample.

VII. Reflectivity of the sample

The reflectivity of the sample with 20° incident angle and 800-nm wavelength, as function of incident polarization states, is measured in Fig. S7. Here we can see the dependence of reflectivity is ~20% on the linear polarization angle (Fig. S7A), and ~10% on the helicity (Fig. S7B). This could be explained by the Fresnel equation, where although the sample is polycrystalline, at the oblique incident cases, the reflectivity for s- and p-component of light are still different. In addition, in Fig. S7B, the left- and right-circularly polarized light is with the same reflectivity, which excludes the circular dichroism of the sample.

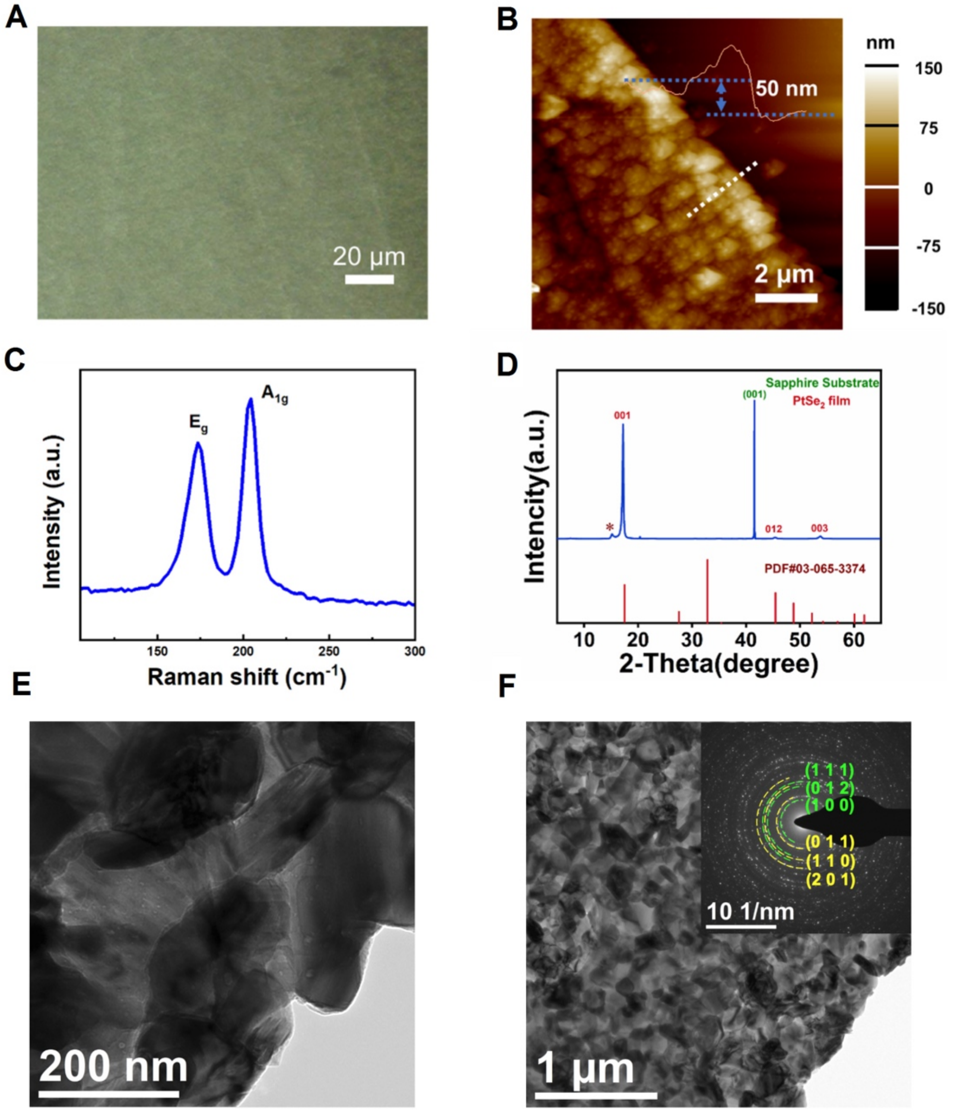

Fig. S1. Sample characterization data of our $PtSe_2$ thin film

(A) Optical image of as-grown $PtSe_2$ film. (B) The corresponding AFM image and height profile. (C) A typical Raman spectrum of the $PtSe_2$ film on SiO_2/Si. (D) XRD pattern of $PtSe_2$ film on sapphire substrate, the "*" symbol was the satellite peak of (001) main peak. (E) TEM image of a 50 nm $PtSe_2$ film, where the grains size is about 200 nm. (F) Local zoom-in of the areas corresponding to (E); the inset in (F) is the SAED which shows the orientation of the grains.

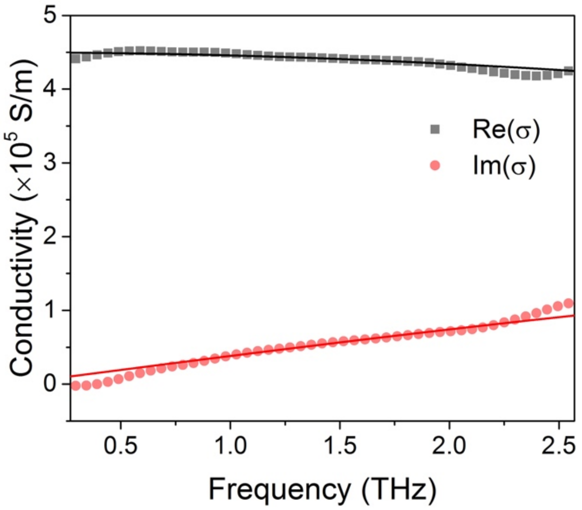

Fig. S2. THz conductivity of $PtSe_2$ thin film

THz conductivity of $PtSe_2$ thin film obtained from THz-TDS measurement at room temperature. The solid curves represent for the Drude model fitting.

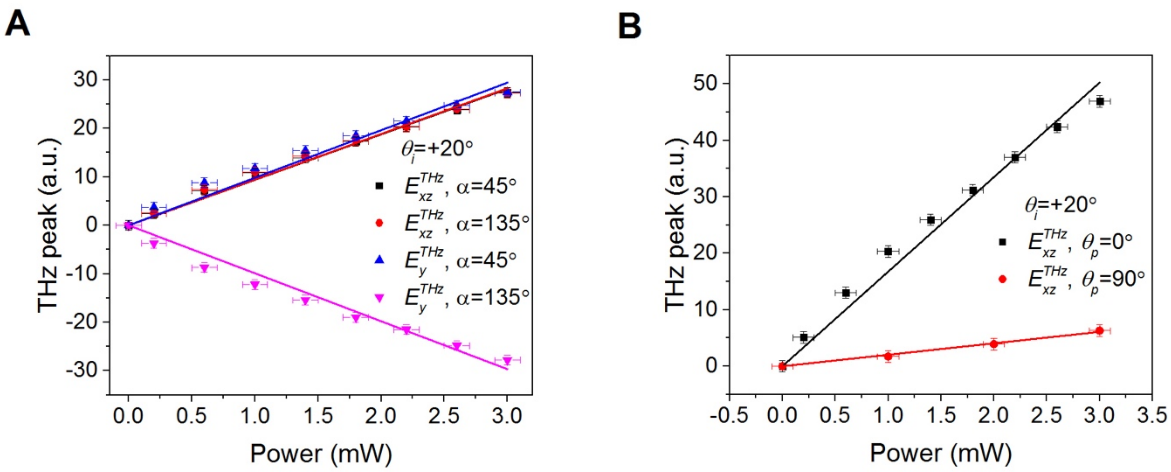

Fig. S3. Pump power dependence of THz emission from $PtSe_2$ thin film with incident pump angle $\boldsymbol{\theta_i = +20°}$

(A) THz peak values of E_x^{THz} and E_y^{THz} components as function of pump power with left ($\alpha = 45°$) and right ($\alpha = 135°$) circularly polarized incident pump. (B) THz peak values of E_y component as a function of pump power with horizontal ($\theta_p = 0°$) and vertical ($\theta_i = 90°$) linear polarized incident pump.

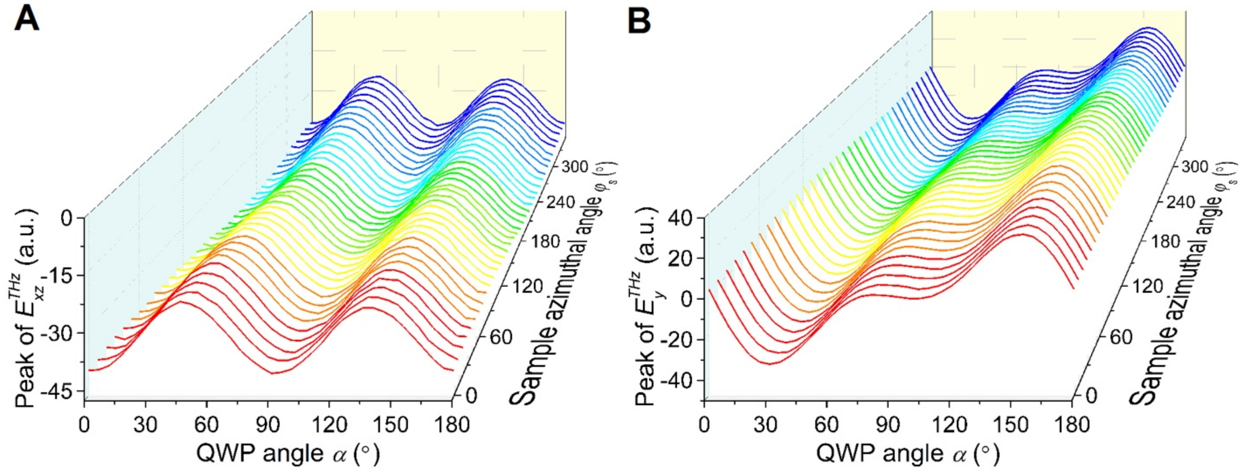

Fig. S4. Extra QWP-angle and azimuthal-angle-dependent THz emission data

(A) and (B) are the emitted THz peak as function of QWP angle α and sample azimuthal angle φ_s for E_x^{THz} and E_y^{THz}, respectively.

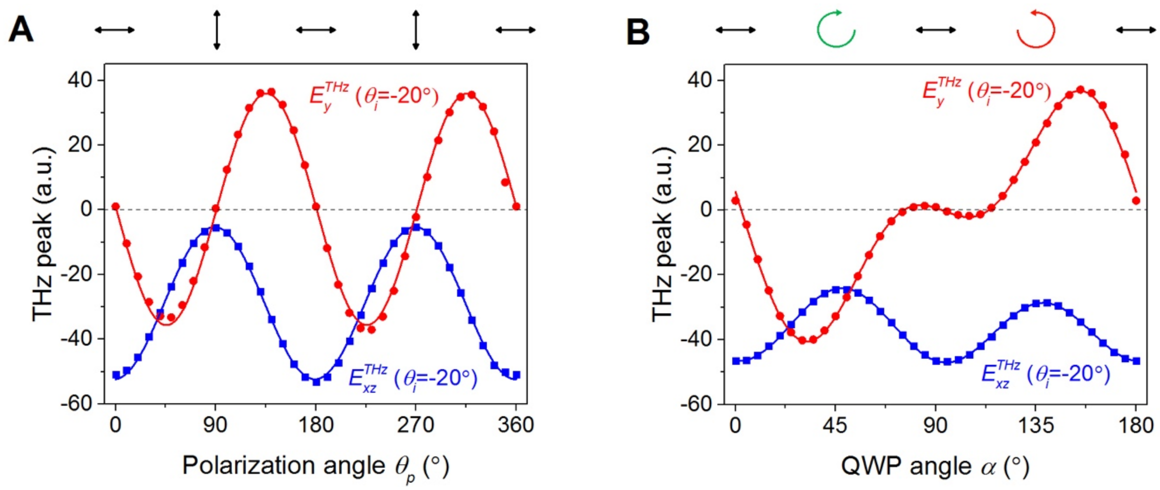

Fig S5. Extra THz emission data of $PtSe_2$ with different polarization states

(A) and (B) are the emitted THz peak as function of polarization angle θ_p and QWP angle α with pump incident angle $\theta_i = -20°$ for E_x^{THz} and E_y^{THz}, respectively.

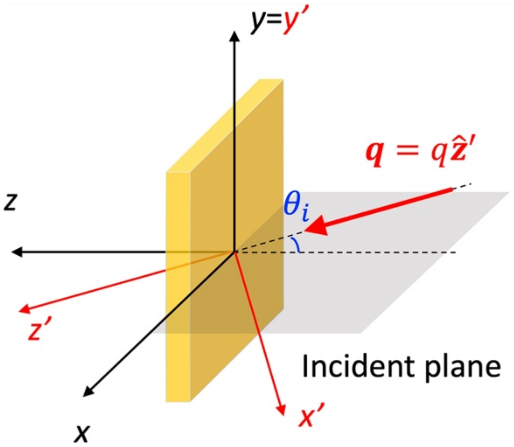

Fig. S6. Schematic of experimental configurations

The sample surface frame is xyz and the lab coordinate frame is $x'y'z'$, and the pump incident angle is θ_i.

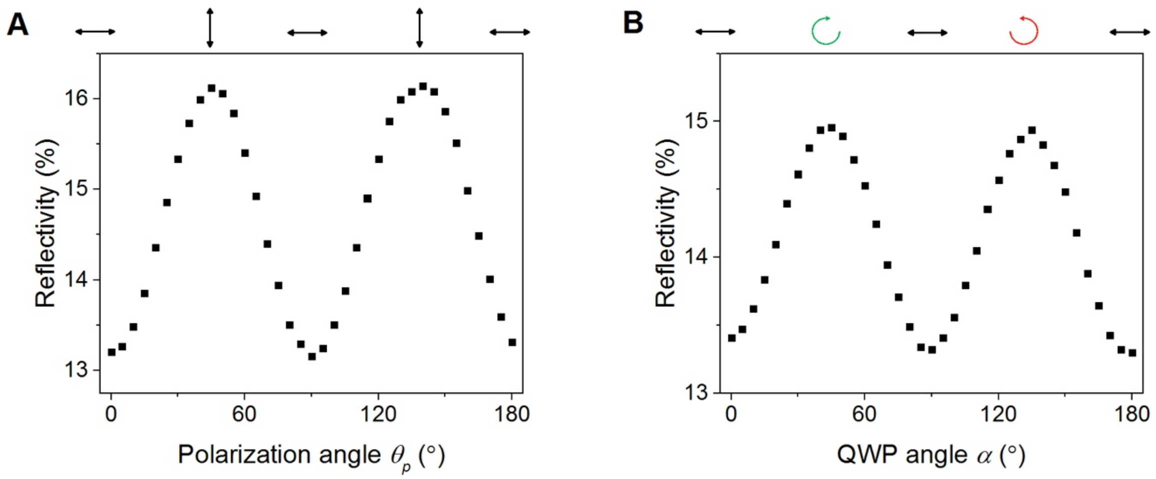

Fig. S7. Reflectivity of $PtSe_2$ thin film with 20° incident angle and 800-nm wavelength
(A) is the linear polarized incident light case, and the polarization angle is tuned by a HWP. (B) is the helicity dependent case, where a QWP is applied to tune the helicity of incident light.

Table S1. Drude model fitting parameters

Sample	Thickness (nm)	$\omega_p/2\pi$ (THz)	$\gamma/2\pi$ (THz)
$PtSe_2$	50	274$\pm$6	9.2$\pm$0.4

REFERENCES AND NOTES

1. T. Morimoto, N. Nagaosa, Topological nature of nonlinear optical effects in solids. *Sci. Adv.* **2**, e1501524 (2016).

2. J. Liu, F. Xia, D. Xiao, F. J. García de Abajo, D. Sun, Semimetals for high-performance photodetection. *Nat. Mater.* **19**, 830–837 (2020).

3. Q. Ma, A. G. Grushin, K. S. Burch, Topology and geometry under the nonlinear electromagnetic spotlight. *Nat. Mater.* **20**, 1601–1614 (2021).

4. L. Wu, S. Patankar, T. Morimoto, N. L. Nair, E. Thewalt, A. Little, J. G. Analytis, J. E. Moore, J. Orenstein, Giant anisotropic nonlinear optical response in transition metal monopnictide Weyl semimetals. *Nat. Phys.* **13**, 350–355 (2017).

5. S. Patankar, L. Wu, B. Lu, M. Rai, J. D. Tran, T. Morimoto, D. E. Parker, A. G. Grushin, N. L. Nair, J. G. Analytis, J. E. Moore, J. Orenstein, D. H. Torchinsky, Resonance-enhanced optical nonlinearity in the Weyl semimetal TaAs. *Phys. Rev. B.* **98**, 165113 (2018).

6. Q. Ma, S.-Y. Xu, C.-K. Chan, C.-L. Zhang, G. Chang, Y. Lin, W. Xie, T. Palacios, H. Lin, S. Jia, P. A. Lee, P. Jarillo-Herrero, N. Gedik, Direct optical detection of Weyl fermion chirality in a topological semimetal. *Nat. Phys.* **13**, 842–847 (2017).

7. G. B. Osterhoudt, L. K. Diebel, M. J. Gray, X. Yang, J. Stanco, X. Huang, B. Shen, N. Ni, P. J. W. Moll, Y. Ran, K. S. Burch, Colossal mid-infrared bulk photovoltaic effect in a type-I Weyl semimetal. *Nat. Mater.* **18**, 471–475 (2019).

8. J. Ma, Q. Gu, Y. Liu, J. Lai, P. Yu, X. Zhuo, Z. Liu, J.-H. Chen, J. Feng, D. Sun, Nonlinear photoresponse of type-II Weyl semimetals. *Nat. Mater.* **18**, 476–481 (2019).

9. Q. Ma, S. Y. Xu, H. Shen, D. MacNeill, V. Fatemi, T. R. Chang, A. M. Mier Valdivia, S. Wu, Z. Du, C. H. Hsu, S. Fang, Q. D. Gibson, K. Watanabe, T. Taniguchi, R. J. Cava, E. Kaxiras, H. Z. Lu, H. Lin, L. Fu, N. Gedik, P. Jarillo-Herrero, Observation of the nonlinear Hall effect under time-reversal-symmetric conditions. *Nature* **565**, 337–342 (2019).

10. D. Kumar, C.-H. Hsu, R. Sharma, T.-R. Chang, P. Yu, J. Wang, G. Eda, G. Liang, H. Yang, Room-temperature nonlinear Hall effect and wireless radiofrequency rectification in Weyl semimetal $TaIrTe_4$. *Nat. Nanotechnol.* **16**, 421–425 (2021).

11. O. O. Shvetsov, V. D. Esin, A. V. Timonina, N. N. Kolesnikov, E. V. Deviatov, Nonlinear hall effect in three-dimensional Weyl and Dirac semimetals. *JETP Lett.* **109**, 715–721 (2019).

12. Y. Gao, S. Kaushik, E. J. Philip, Z. Li, Y. Qin, Y. P. Liu, W. L. Zhang, Y. L. Su, X. Chen, H. Weng, D. E. Kharzeev, M. K. Liu, J. Qi, Chiral terahertz wave emission from the Weyl semimetal TaAs. *Nat. Commun.* **11**, 720 (2020).

13. N. Sirica, R. I. Tobey, L. X. Zhao, G. F. Chen, B. Xu, R. Yang, B. Shen, D. A. Yarotski, P. Bowlan, S. A. Trugman, J.-X. Zhu, Y. M. Dai, A. K. Azad, N. Ni, X. G. Qiu, A. J. Taylor, R. P. Prasankumar, Tracking ultrafast photocurrents in the Weyl semimetal TaAs using THz emission spectroscopy. *Phys. Rev. Lett.* **122**, 197401 (2019).

14. L. Luo, D. Cheng, B. Song, L. L. Wang, C. Vaswani, P. M. Lozano, G. Gu, C. Huang, R. H. J. Kim, Z. Liu, J. M. Park, Y. Yao, K. Ho, I. E. Perakis, Q. Li, J. Wang, A light-induced phononic symmetry switch and giant dissipationless topological photocurrent in $ZrTe_5$. *Nat. Mater.* **20**, 329–334 (2021).

15. X. Zhou, B. Song, X. Chen, Y. You, S. Ruan, H. Bai, W. Zhang, G. Ma, J. Yao, F. Pan, Z. Jin, C. Song, Orientation-dependent THz emission in noncollinear antiferromagnetic Mn_3Sn and Mn_3Sn-based heterostructures. *Appl. Phys. Lett.* **115**, 182402 (2019).

16. L. Ding, X. Luo, L. Cheng, M. Thway, J. Song, S. J. Chua, E. E. M. Chia, J. Teng, Electrically and thermally tunable smooth silicon metasurfaces for broadband terahertz antireflection. *Adv. Opt. Mater.* **6**, 1800928 (2018).

17. M. Chen, K. Lee, J. Li, L. Cheng, Q. Wang, K. Cai, E. E. M. Chia, H. Chang, H. Yang, Anisotropic picosecond spin-photocurrent from Weyl semimetal WTe_2. *ACS Nano* **14**, 3539–3545 (2020).

18. W. Lu, J. Ling, F. Xiu, D. Sun, Terahertz probe of photoexcited carrier dynamics in the Dirac semimetal Cd_3As_2. *Phys. Rev. B* **98**, 104310 (2018).

19. J. W. McIver, D. Hsieh, H. Steinberg, P. Jarillo-Herrero, N. Gedik, Control over topological insulator photocurrents with light polarization. *Nat. Nanotechnol.* **7**, 96–100 (2012).

20. J. Wei, C. Xu, B. Dong, C.-W. Qiu, C. Lee, Mid-infrared semimetal polarization detectors with configurable polarity transition. *Nat. Photonics* **15**, 614–621 (2021).

21. L. Shi, D. Zhang, K. Chang, J. C. W. Song, Geometric photon-drag effect and nonlinear shift current in centrosymmetric crystals. *Phys. Rev. Lett.* **126**, 197402 (2021).

22. H. Kurosawa, K. Sawada, S. Ohno, Photon drag effect due to berry curvature. *Phys. Rev. Lett.* **117**, 083901 (2016).

23. V. A. Shalygin, M. D. Moldavskaya, S. N. Danilov, I. I. Farbshtein, L. E. Golub, Circular photon drag effect in bulk tellurium. *Phys. Rev. B* **93**, 045207 (2016).

24. L. Zhu, Y. Huang, Z. Yao, B. Quan, L. Zhang, J. Li, C. Gu, X. Xu, Z. Ren, Enhanced polarization-sensitive terahertz emission from vertically grown graphene by a dynamical photon drag effect. *Nanoscale* **9**, 10301–10311 (2017).

25. L. Zhu, Z. Yao, Y. Huang, C. He, B. Quan, J. Li, C. Gu, X. Xu, Z. Ren, Circular-photon-drag-effect-induced elliptically polarized terahertz emission from vertically grown graphene. *Phys. Rev. Appl.* **12**, 044063 (2019).

26. C. Jiang, V. A. Shalygin, V. Y. Panevin, S. N. Danilov, M. M. Glazov, R. Yakimova, S. Lara-Avila, S. Kubatkin, S. D. Ganichev, Helicity-dependent photocurrents in graphene layers excited by midinfrared radiation of a CO_2 laser. *Phys. Rev. B* **84**, 125429 (2011).

27. V. A. Shalygin, H. Diehl, C. Hoffmann, S. N. Danilov, T. Herrle, S. A. Tarasenko, D. Schuh, C. Gerl, W. Wegscheider, W. Prettl, S. D. Ganichev, Spin photocurrents and the circular photon drag effect in (110)-grown quantum well structures. *JETP Lett.* **84**, 570–576 (2007).

28. J. Maysonnave, S. Huppert, F. Wang, S. Maero, C. Berger, W. de Heer, T. B. Norris, L. A. De Vaulchier, S. Dhillon, J. Tignon, R. Ferreira, J. Mangeney, Terahertz generation by dynamical photon drag effect in graphene excited by femtosecond optical pulses. *Nano Lett.* **14**, 5797–5802 (2014).

29. J. Karch, P. Olbrich, M. Schmalzbauer, C. Zoth, C. Brinsteiner, M. Fehrenbacher, U. Wurstbauer, M. M. Glazov, S. A. Tarasenko, E. L. Ivchenko, D. Weiss, J. Eroms, R. Yakimova, S. Lara-Avila, S. Kubatkin, S. D. Ganichev, Dynamic hall effect driven by circularly polarized light in a graphene layer. *Phys. Rev. Lett.* **105**, 227402 (2010).

30. Z.-X. Zhang, L.-H. Zeng, X.-W. Tong, Y. Gao, C. Xie, Y. H. Tsang, L.-B. Luo, Y.-C. Wu, Ultrafast, self-driven, and air-stable photodetectors based on multilayer $PtSe_2$/perovskite heterojunctions. *J. Phys. Chem. Lett.* **9**, 1185–1194 (2018).

31. X. Yu, P. Yu, D. Wu, B. Singh, Q. Zeng, H. Lin, W. Zhou, J. Lin, K. Suenaga, Z. Liu, Q. J. Wang, Atomically thin noble metal dichalcogenide: A broadband mid-infrared semiconductor. *Nat. Commun.* **9**, 1545 (2018).

32. L. Wang, S. Zhang, N. McEvoy, Y. Sun, J. Huang, Y. Xie, N. Dong, X. Zhang, I. M. Kislyakov, J. Nunzi, L. Zhang, J. Wang, Nonlinear optical signatures of the transition from semiconductor to semimetal in $PtSe_2$. *Laser Photon. Rev.* **13**, 1900052 (2019).

33. K. Zhang, M. Yan, H. Zhang, H. Huang, M. Arita, Z. Sun, W. Duan, Y. Wu, S. Zhou, Experimental evidence for type-II Dirac semimetal in $PtSe_2$. *Phys. Rev. B* **96**, 125102 (2017).

34. M. S. Bahramy, O. J. Clark, B.-J. Yang, J. Feng, L. Bawden, J. M. Riley, I. Marković, F. Mazzola, V. Sunko, D. Biswas, S. P. Cooil, M. Jorge, J. W. Wells, M. Leandersson, T. Balasubramanian, J. Fujii, I. Vobornik, J. E. Rault, T. K. Kim, M. Hoesch, K. Okawa, M. Asakawa, T. Sasagawa, T. Eknapakul, W. Meevasana, P. D. C. King, Ubiquitous formation of bulk Dirac cones and topological surface states from a single orbital manifold in transition-metal dichalcogenides. *Nat. Mater.* **17**, 21–28 (2018).

35. Y. Huang, Z. Yao, C. He, L. Zhu, L. Zhang, J. Bai, X. Xu, Terahertz surface and interface emission spectroscopy for advanced materials. *J. Phys. Condens. Matter* **31**, 153001 (2019).

36. Z. Fang, H. Wang, X. Wu, S. Shan, C. Wang, H. Zhao, C. Xia, T. Nie, J. Miao, C. Zhang, W. Zhao, L. Wang, Nonlinear terahertz emission in the three-dimensional topological insulator Bi_2Te_3 by terahertz emission spectroscopy. *Appl. Phys. Lett.* **115**, 191102 (2019).

37. S. Lai, H. Liu, Z. Zhang, J. Zhao, X. Feng, N. Wang, C. Tang, Y. Liu, K. S. Novoselov, S. A. Yang, W. Gao, Third-order nonlinear Hall effect induced by the Berry-connection polarizability tensor. *Nat. Nanotechnol.* **16**, 869–873 (2021).

38. V. Apostolopoulos, M. E. Barnes, THz emitters based on the photo-Dember effect. *J. Phys. D Appl. Phys.* **47**, 374002 (2014).

39. W. Lu, Z. Fan, Y. Yang, J. Ma, J. Lai, X. Song, X. Zhuo, Z. Xu, J. Liu, X. Hu, S. Zhou, F. Xiu, J. Cheng, D. Sun, Ultrafast photothermoelectric effect in Dirac semimetallic Cd_3As_2 revealed by terahertz emission. *Nat. Commun.* **13**, 1623 (2022).

40. Y. Xiong, L. Shi, J. C. W. Song, Unblocking time-reversal forbidden photocurrents in non-magnetic materials. arXiv:2108.07823 [cond-mat.mes-hall] (17 August 2021).

41. J. Ahn, G.-Y. Guo, N. Nagaosa, Low-frequency divergence and quantum geometry of the bulk photovoltaic effect in topological semimetals. *Phys. Rev. X* **10**, 041041 (2020).

42. J. E. Sipe, A. I. Shkrebtii, Second-order optical response in semiconductors. *Phys. Rev. B* **61**, 5337–5352 (2000).

43. F. de Juan, A. G. Grushin, T. Morimoto, J. E. Moore, Quantized circular photogalvanic effect in Weyl semimetals. *Nat. Commun.* **8**, 15995 (2017).

44. R. von Baltz, W. Kraut, Theory of the bulk photovoltaic effect in pure crystals. *Phys. Rev. B* **23**, 5590–5596 (1981).

45. H. Huang, S. Zhou, W. Duan, Type-II Dirac fermions in the $PtSe_2$ class of transition metal dichalcogenides. *Phys. Rev. B* **94**, 121117 (2016).

46. A. M. Danishevskii, A. A. Kastalskii, S. M. Ryvkin, Dragging of free carriers by photons in direct interband transitions in semiconductors. *Sov. Phys. JETP* **31**, 292–295 (1970).

47. L. Cheng, X. Wang, W. Yang, J. Chai, M. Yang, M. Chen, Y. Wu, X. Chen, D. Chi, K. E. J. Goh, J.-X. Zhu, H. Sun, S. Wang, J. C. W. Song, M. Battiato, H. Yang, E. E. M. Chia, Far out-of-equilibrium spin populations trigger giant spin injection into atomically thin MoS_2. *Nat. Phys.* **15**, 347–351 (2019).

48. X. Wang, L. Cheng, D. Zhu, Y. Wu, M. Chen, Y. Wang, D. Zhao, C. B. Boothroyd, Y. M. Lam, J.-X. Zhu, M. Battiato, J. C. W. Song, H. Yang, E. E. M. Chia, Ultrafast spin-to-charge conversion at the surface of topological insulator thin films. *Adv. Mater.* **30**, 1802356 (2018).

49. N. Laman, D. Grischkowsky, Terahertz conductivity of thin metal films. *Appl. Phys. Lett.* **93**, 051105 (2008).

50. J. Lourembam, A. Srivastava, C. La-o-vorakiat, H. Rotella, T. Venkatesan, E. E. M. Chia, New insights into the diverse electronic phases of a novel vanadium dioxide polymorph: A terahertz spectroscopy study. *Sci. Rep.* **5**, 9182 (2015).

51. Y. Onishi, Z. Ren, M. Novak, K. Segawa, Y. Ando, K. Tanaka, Instantaneous photon drag currents in topological insulators. arXiv:1403.2492 [cond-mat.mtrl-sci] (10 Sep 2014).

52. T. Seifert, S. Jaiswal, U. Martens, J. Hannegan, L. Braun, P. Maldonado, F. Freimuth, A. Kronenberg, J. Henrizi, I. Radu, E. Beaurepaire, Y. Mokrousov, P. M. Oppeneer, M. Jourdan, G. Jakob, D. Turchinovich, L. M. Hayden, M. Wolf, M. Münzenberg, M. Kläui, T. Kampfrath, Efficient metallic spintronic emitters of ultrabroadband terahertz radiation. *Nat. Photonics* **10**, 483–488 (2016).

53. Y. Li, Y. Xia, S. A. Ekahana, N. Kumar, J. Jiang, L. Yang, C. Chen, C. Liu, B. Yan, C. Felser, G. Li, Z. Liu, Y. Chen, Topological origin of the type-II Dirac fermions in $PtSe_2$. *Phys. Rev. Mater.* **1**, 074202 (2017).

54. R. W. Boyd, *Nonlinear Optics* (Elsevier, 2003).

55. C. Kittel, *Introduction to Solid State Physics* (Wiley, ed. 8, 2004).
